# Complete genome sequence of *Microbacterium paraoxydans* phage Damascus

**DOI:** 10.1128/mra.01287-23

**Published:** 2024-04-16

**Authors:** Julisa M. Bearhart, Jenna L. Bethke, Cassie S. Christian, Faith N. Cour, Karleigh R. Creasey, Emily J. Crowe, Julia G. Dahl, Lindsey A. Hanson, Abby L. Jaecks, Vincent A. Lamantia, Mercedes Madison, Autumn L. Roskowiak, Justin D. Scheberl, Bekkah M. VanEperen, Morgan E. Wurst, Karen K. Klyczek

**Affiliations:** 1 Department of Biology, University of Wisconsin-River Falls, River Falls, Wisconsin, USA; Loyola University Chicago, Chicago, Illinois, USA

**Keywords:** phage, bacteriophage genomics, actinobacteriophage

## Abstract

Phage Damascus was isolated from soil in northwestern Wisconsin using *Microbacterium paraoxydans* as the host. The Damascus genome is 56,477 bp with 3′ single-stranded overhangs and 56.5% G+C content. Damascus was assigned to cluster EL and shares 42.6%–91.7% gene content with the three other phages in this cluster.

## ANNOUNCEMENT

**Fig 1:**
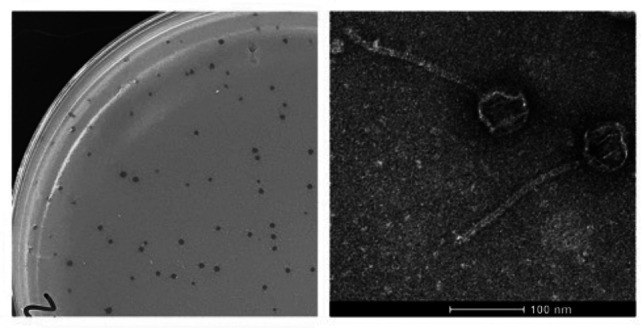
Characteristics of phage Damascus. (Left) Representative plaques formed by phage Damascus on *Microbacterium paraoxydans*; (right) high-titer lysates were placed on Formvar-coated grids, negatively stained with 1% uranyl acetate (2) and imaged using a FEI Tecnai Spirit BioTwin transmission electron microscope at 120 kV. Damascus has an average head diameter of 62 nm and tail length of 196 nm (*n* = 6).

Comparative analysis of the genomes of phages infecting a single host genus, such as *Microbacterium*, has provided important insights into bacteriophage evolution and diversity (1). Here, we report the genome sequence of phage Damascus, isolated on *Microbacterium paraoxydans* NRRL B-14843. Damascus was isolated from soil collected 12 September 2022 in Amery, WI (45.311936 N, 92.366226 W) using standard procedures (2). Briefly, soil was suspended in peptone-yeast extract-calcium (PYCa) liquid medium and incubated with shaking at 250  rpm for 2 h at 30°C. The wash was collected by centrifugation and filtration (0.22-μm pore size), and the filtrate was inoculated with an overnight culture of *Microbacterium paraoxydans*. Following incubation with shaking for 48 h at 30°C, the culture was filtered, and the filtrate was plated in PYCa top agar with *Microbacterium paraoxydans* using three rounds of plaque purification. Plates were incubated for 24 h at 30°C. Plaques were clear and 1–2 mm in diameter. Negative stain transmission electron microscopy revealed that Damascus has siphoviral morphology, with an isometric capsid and a long, flexible tail (Fig. 1).

Double-stranded DNA was isolated from phage lysate using the Promega Wizard DNA cleanup system, and a sequencing library was prepared using the NEB Ultra II kit. Sequencing was performed using an Illumina MiSeq system (v.3 reagents), yielding 471,080 150-bp single-end reads (1,197-fold genome coverage). Raw reads were assembled using Newbler (v.2.9), and completeness was verified using Consed (v.29.0) (3). The Damascus genome is 56,477 bp with nine-base 3′ single-stranded overhangs (CGCGTCACT) and 56.6% G+C content.

The genome was annotated using DNA Master (http://cobamide2.bio.pitt.edu), PECAAN (https://discover.kbrinsgd.org), GLIMMER (v.3.02) (4), GeneMark (v.2.5) (5), Starterator (v.1.1) (http://phages.wustl.edu/starterator/), and Phamerator (6). Gene functions were predicted using BLASTp using the National Center for Biotechnology Information (NCBI) nonredundant database (7) and Actinobacteriophage Database (8), HHpred (9) using the PDB mmCIF70, Pfam-A, and NCBI Conserved Domain databases, and DeepTMHMM (10). Genes encoding tRNAs were identified using ARAGORN (v.1.2.38) (11) and tRNAscan-SE (v.3.0) (12). Default settings were used for all programs. Annotation identified 82 protein-coding genes and no tRNA genes. We were able to assign predicted functions for 23 gene products (28%). Two gene products (gp40 and gp66) have no homologs in the database (8). Damascus is predicted to have a lytic life cycle based on the absence of genes associated with lysogeny.

Damascus was assigned to cluster EL, based on gene content similarity (GCS) of 35% or higher with phages in the Actinobacteriophage Database (8), as described (13). Within cluster EL, Damascus shares 91.7% GCS with DizzyRudy (MK814756), which was isolated on the same strain of *M. paraoxydans*, but only 72.4% and 42.6% with Camille (MH153800) and Count (MH153801), respectively, isolated on *Microbacterium aerolatum* B-24229. Damascus shares notable genome features with other cluster EL phages and with phages in several clusters (AM, AU, AW, BI CC, and DJ) isolated on different host genera (1). These features include a fused major capsid subunit and capsid maturation protease (gp14), two major tail proteins (gp17 and gp19), and relatively low percent G+C content compared to the host bacteria (56.5% for Damascus vs 67% for *Microbacterium* hosts) (1).

## Data Availability

Damascus is available at GenBank with accession number OQ995438 and Sequence Read Archive accession number SRX19690868.

## References

[1] Jacobs-Sera D, Abad LA, Alvey RM, Anders KR, Aull HG, Bhalla SS, Blumer LS, Bollivar DW, Bonilla JA, Butela KA, et al.. 2020. Genomic diversity of bacteriophages infecting Microbacterium spp. PLoS ONE 15:e0234636. doi:10.1371/journal.pone.0234636 32555720 PMC7302621

[2] Poxleitner M, Pope W, Jacobs-Sera D, Sivanathan V, Hatfull GF. 2018. HHMI SEA-PHAGES phage discovery guide. Available from: https://seaphagesphagediscoveryguide.helpdocsonline.com/home

[3] Russell DA. 2018. Sequencing, assembling, and finishing complete bacteriophage genomes. Methods Mol Biol 1681:109–125. doi:10.1007/978-1-4939-7343-9_9 29134591

[4] Delcher AL, Bratke KA, Powers EC, Salzberg SL. 2007. Identifying bacterial genes and endosymbiont DNA with glimmer. Bioinformatics 23:673–679. doi:10.1093/bioinformatics/btm009 17237039 PMC2387122

[5] Besemer J, Borodovsky M. 2005. GeneMark: web software for gene finding in prokaryotes, eukaryotes and viruses. Nucleic Acids Res 33:W451–W454. doi:10.1093/nar/gki487 15980510 PMC1160247

[6] Cresawn SG, Bogel M, Day N, Jacobs-Sera D, Hendrix RW, Hatfull GF. 2011. Phamerator: a bioinformatic tool for comparative bacteriophage genomics. BMC Bioinformatics 12:395. doi:10.1186/1471-2105-12-395 21991981 PMC3233612

[7] Altschul SF, Gish W, Miller W, Myers EW, Lipman DJ. 1990. Basic local alignment search tool. J Mol Biol 215:403–410. doi:10.1016/S0022-2836(05)80360-2 2231712

[8] Russell DA, Hatfull GF. 2017. PhagesDB: the actinobacteriophage database. Bioinformatics 33:784–786. doi:10.1093/bioinformatics/btw711 28365761 PMC5860397

[9] Söding J, Biegert A, Lupas AN. 2005. The HHpred interactive server for protein homology detection and structure prediction. Nucleic Acids Res 33:W244–8. doi:10.1093/nar/gki408 15980461 PMC1160169

[10] Hallgren J, Tsirigos KD, Pedersen MD, Almagro Armenteros JJ, Marcatili P, Nielsen H, Krogh A, Winther O. 2022. DeepTMHMM predicts alpha and beta transmembrane proteins using deep neural networks. bioRxiv. doi:10.1101/2022.04.08.487609

[11] Laslett D, Canback B. 2004. ARAGORN, a program to detect tRNA genes and tmRNA genes in nucleotide sequences. Nucleic Acids Res 32:11–16. doi:10.1093/nar/gkh152 14704338 PMC373265

[12] Lowe TM, Eddy SR. 1997. tRNAscan-SE: a program for improved detection of transfer RNA genes in genomic sequence. Nucleic Acids Res 25:955–964. doi:10.1093/nar/25.5.955 9023104 PMC146525

[13] Pope WH, Mavrich TN, Garlena RA, Guerrero-Bustamante CA, Jacobs-Sera D, Montgomery MT, Russell DA, Warner MH, Hatfull GF, Science Education Alliance-Phage Hunters Advancing Genomics and Evolutionary Science (SEA-PHAGES). 2017. Bacteriophages of Gordonia spp. display a spectrum of diversity and genetic relationships. mBio 8:e01069-17. doi:10.1128/mBio.01069-17 28811342 PMC5559632

